# Contexto de cuidado del enfermo renal crónico: conceptos existenciales y humanísticos*

**DOI:** 10.15649/cuidarte.3006

**Published:** 2023-09-09

**Authors:** Carolina Giordani da Silva, Maria da Grata Oliveira Crossetti, Maravilla Giménez Fernández, Janaína dos Santos Prates

**Affiliations:** 1 Universidade Federal do Mato Grosso, Mato Grosso, Brasil. Email: carol.giordani@gmail.com Universidade Federal do Mato Grosso Universidade Federal do Mato Grosso Mato Grosso Brazil carol.giordani@gmail.com; 2 Universidade Federal do Rio Grande do Sul, Rio Grande do Sul, Brasil. Email: mgcrossetti@gmail.com Universidade Federal do Rio Grande do Sul Universidade Federal do Rio Grande do Sul Rio Grande do Sul Brazil mgcrossetti@gmail.com; 3 Universidad Católica San Antonio de Murcia, Murcia, España. Email: mgimenez@ucam.edu Universidad Católica San Antonio de Murcia Universidad Católica San Antonio de Murcia Murcia Spain mgimenez@ucam.edu; 4 Universidade Federal do Rio Grande do Sul, Rio Grande do Sul, Brasil. Email: janainaprates1997@outlook.com Universidade Federal do Rio Grande do Sul Universidade Federal do Rio Grande do Sul Rio Grande do Sul Brazil janainaprates1997@outlook.com

**Keywords:** Ambiente, Teorías de enfermería, Existencialismo, Teoria fundamentada, Insuficiencia renal crónica, Environment, Nursing theory, Existentialism, Grounded theory, Chronic kidney failure, Ambiente, Teorias de Enfermagem, Existencialismo, Teoria Fundamentada, Insuficiencia Renal Crónica

## Abstract

**Introducción:**

La Enfermedad Renal Crónica es la pérdida irreversible de la función renal, requierendo terapia renal sustitutiva, basada en la evidencia científica, con excelentes resultados, pero que descuida las complejidades del ambiente asistencial, como los sentimientos, considerados evidencia cualitativa, inherente a la dimensión existencial del individuo.

**Objetivo:**

develar conceptos existenciales en el contexto del cuidado del ser-en-el- mundo de la enfermedad renal crónica desde la perspectiva de la teoría humanista de enfermería.

**Materiales y Métodos:**

estudio cualitativo del tipo teoría fundamentada, desarrollado en un hospital universitario del sur de Brasil. La muestra fue 10 profesionales de enfermería y 10 pacientes en terapia renal sustitutiva. Los datos fueron recolectados entre enero de 2020 y enero de 2021. El análisis de los datos se basó en la filosofía existencialista y en la gran teoría humanística de la enfermería.

**Resultados:**

Se develó una categoría - Contexto de cuidado del ser-en el-mundo de la enfermedad renal crónica, dos subcategorías, ambiente y temporalidad, que dio origen a cinco conceptos, ambiente, adaptación a la realidad, establecer un espacio de convivencia, temporalidad y convertirse en rutina, y una suposición, el paso del tiempo.

**Discusión:**

Contexto de Cuidado es un ambiente que es un espacio vivo, dinámico, de inter-relaciones entre los seres-de-este-mundo.

**Conclusión:**

Se entiende que la enfermería, como ciencia teórico-práctica, necesita referentes teóricos que orienten la práctica clínica hacia un cuidado que contemple las dimensiones biológicas, espirituales y existenciales del ser, haciendo más efectivo este cuidado, considerando el ambiente, que influye en la adaptación y adherencia al tratamiento.

## Abstract (en)

**Introduction:**

Chronic kidney disease is the irreversible loss of kidney function and requires renal replacement therapy, which has excellent results based on scientific evidence, but neglects the complexities of the care environment, such as feelings, considered qualitative evidence inherent to the existential dimension of the individual.

**Objective:**

To unveil existential concepts in the context of care for the being-in-the-world of chronic kidney disease from the perspective of humanistic nursing theory.

**Materials and methods:**

Qualitative study using Grounded Theory. The study was conducted in a university hospital in southern Brazil. The sample consisted of 10 nursing professionals and 10 patients on renal replacement therapy. Data were collected between January 2020 and January 2021 through a semi-structured interview conducted virtually using the Zoom application. Data analysis was based on existentialist philosophy and the grand humanistic nursing theory.

**Results:**

One category was revealed (context of care of chronic kidney disease's being-in-the-world), and two subcategories (environment and temporality), which gave rise to five concepts (environment, adaptation to reality, establishing a living space, temporality, and becoming routine) and one assumption (the passage of time).

**Discussion:**

The context of care is an environment that is a living, dynamic space of interrelationships between beings of this world.

**Conclusion:**

It is understood that nursing, as a theoretical-practical science, needs theoretical reference points that guide clinical a type of care that considers the biological, spiritual, and existential dimensions of the being, making this care more effective, considering the environment, which influences adaptation and adherence to treatment.

## Resumo

**Introdujo:**

A Doenga Renal Crónica é a perda irreversível da fungáo renal, necessitando de terapia renal substitutiva, baseada em evidencias científicas, com excelentes resultados, mas que negligencia as complexidades do ambiente de cuidado, como os sentimentos, consideradas evidencias qualitativas, inerentes a dimensáo existencial do individuo.

**Objetivo:**

revelar concepgóes existenciais no contexto do cuidado ao ser-no-mundo da doenga renal crónica na perspectiva da teoria humanista de enfermagem.

**Materiais e Métodos:**

estudo qualitativo do tipo Grounded Theory, desenvolvido em um hospital universitário do sul do Brasil. A amostra foi composta por 10 profissionais de enfermagem e 10 pacientes em terapia renal substitutiva. Os dados foram coletados entre janeiro de 2020 e janeiro de 2021, por meio de entrevista semiestruturada, realizada virtualmente por meio do aplicativo Zoom. A análise dos dados baseou-se na filosofia existencialista e na grande teoria humanística da enfermagem.

**Resultados:**

Emergiu uma categoria - Contexto de cuidar do ser-no-mundo da doenga renal crónica, duas subcategorias, ambiente e temporalidade, que deram origem a cinco conceitos: ambiente, adaptagáo a realidade, estabelecendo um espago de convivencia, temporalidade e tornar-se rotina, e uma suposigáo, a passagem do tempo.

**Discussao:**

Contexto de Cuidado é um ambiente que é um espago vivo, dinámico, de inter- relagóes entre seres-deste-mundo.

**Conclusao:**

Compreende-se que a enfermagem, como ciencia teórico-prática, necessita de referenciais teóricos que orientem a prática clínica para um cuidado que contemple as dimensóes biológica, espiritual e existencial do ser, tornando esse cuidado mais efetivo, considerando o ambiente, que influencia na adaptagáo e adesáo ao tratamento.

## Introducción

La Enfermedad Renal Crónica (ERC) es un cambio en la estructura o función de los riñones durante más de tres meses, además de la pérdida progresiva e irreversible de la función renal, contemplada en las Enfermedades Crónicas No Transmisibles (ECNT) que, en los casos más avanzados, etapa 5 o de diálisis, requiere Terapia Sustitutiva Renal (TRS) para mantener la vida^1^^,^^2^.

En Brasil, el último censo realizado en 2021 por la Sociedad Brasileña de Nefrología estimó que el número de personas con ERC en diálisis, en etapa 5, llegó a 148.363, con un aumento promedio de 3.584 pacientes (2,5%) en comparación con el año pasado^3^. El TRS predominante para la ERC es la hemodiálisis, con una prevalencia de hasta el 92% de los pacientes, seguida de la diálisis peritoneal y el trasplante renal. Las TRS son la única posibilidad de supervivencia de los pacientes, pero traen consigo una serie de cambios inherentes a la dimensión física de estos y a su condición existencial.

La condición existencial se refiere a la unicidad del ser, propia a la subjetividad de cada individuo, que se manifiesta en la vida cotidiana con formas de expresión que pueden ser auténticas, únicas y singulares, o inauténticas, inadecuadas e impersonales. Desde una óptica existencial, el ser humano tiene la capacidad de ver, significar y apropiarse de las cosas del mundo desde su propia perspectiva ^4^.

En el mundo de la ERC, esta condición existencial está relacionada con los sentimientos expresados por los pacientes, reconocidos como evidencias cualitativas en el contexto del cuidado y que obliga al equipo de enfermería a atender todas estas necesidades, físicas y existenciales, para brindar cuidados de calidad, efectivos y seguros, minimizando el riesgo para el paciente y el profesional^5^. Se entiende que el contexto del cuidado es el espacio en que el ser-paciente y el ser-enfermero, aquí representados por el equipo de enfermería, coexisten interactuando y compartiendo experiencias y sentimientos que impactan en la condición de ser de cada uno y en la calidad del tratamiento ofrecido allí.

En este sentido, el ambiente de cuidado se caracteriza como un lugar que reúne aspectos físicos, biológicos, científicos, culturales, sociales, económicos, entre otros, en un entramado de relaciones donde se hace difícil excluir o aislar partes, proporcionado el establecimiento de las relaciones de cuidado en situaciones vitales que involucran el proceso salud-enfermedad-atención^6^. Por lo tanto, es necesario comprender el contexto del cuidado más allá de un simple espacio físico en el que se desarrolla la práctica del cuidado de enfermería, lo que está en consonancia con la teoría humanista de la enfermería^7^ que al describir la enfermería como un encuentro entre dos seres en una especie de diálogo vivo, refuerza la influencia del entorno en este encuentro.

Por lo tanto, en la teoría humanista de enfermería el ambiente físico puede servir para facilitar o dificultar el diálogo de enfermería, sin embargo, la experiencia personal del espacio puede ser aún más importante^7^. En esta teoría, el contexto de cuidado es entendido como un tipo de espacio vivo, personalizado, un lugar donde uno se siente perteneciente o bien, del cual uno no se siente parte. Lo que significa que una enfermera y un paciente pueden estar juntos em un lugar y sin embargo, uno sentirse como en casa y el otro no^7^.

Así, en el mundo de la ERC, en el que los encuentros de cuidado ocurren periódicamente, es necesario comprender el contexto del cuidado en su dimensión existencial para que el ser-enfermero pueda facilitar la adhesión del ser-paciente a su TRS, contribuyendo a que se sienta parte de este mundo, y así poder apropiarse de sí mismo y de las cosas del mundo de la ERC, recuperando em la medida de lo posible su autonomía y mejorando su calidad de vida.

En ese sentido, este estudio tuvo como objetivo revelar elementos conceptuales existenciales presentes en el contexto del cuidado por el ser-en-el-mundo de la enfermedad renal crónica en la perspectiva de la teoría humanista de enfermería^7^^-^^8^.

## Materiales y Métodos

Se trata de un estudio exploratorio-descriptivo, con abordaje cualitativo^9^, con el referencial metodológico de la teoría fundamentada (GT)^10^.

El estudio tuvo lugar en un hospital universitario de Rio Grande do Sul, Brasil, en el Servicio de Nefrología, que atiende a pacientes renales agudos o crónicos que necesitan TRS.

La población de estudio estuvo constituida por enfermeros y técnicos de enfermería que actúan en el Servicio de Nefrología y pacientes que se encontraban con algún tipo de TRS, la muestra fue definida por conveniencia, mediante la invitación a participar en el estudio.

La muestra inicial estuvo conformada por 5 enfermeros, 3 técnicos de enfermería y 9 pacientes. Sin embargo, de acuerdo con el muestreo teórico, en atención al referencial metodológico 10, a medida que los datos fueron recolectados y los conceptos teóricos ganaron densidad, y tras la constante comparación, uso de memorandos y pensamiento inductivo-deductivo, surgieron hipótesis, orientando el tamaño muestral que al final fue de 7 enfermeros, 3 técnicos de enfermería y 10 pacientes. Esta cantidad se definió desde el momento en que la información de los participantes no proporcionaba datos relevantes hacia nuevas categorizaciones y formulación de nuevos conceptos teóricos.

Los criterios de inclusión fueron enfermeros y técnicos de enfermería con al menos un año de experiencia en la institución donde se realizó el estudio, presentes durante el período de recolección de datos y pacientes que estuvieran realizando algún tipo de TRS y que pudieran responder a la entrevista. Los criterios de exclusión fueron enfermeros y técnicos de enfermería que no estuvieran trabajando en el cuidado durante el período de la investigación, así como pacientes que tuvieran un déficit cognitivo o neurológico que no les permitiera responder a la investigación y tuvieran dificultades con el uso de tecnologías, como teléfono móvil y/o ordenador para entrevistas virtuales.

La recolección y análisis de datos se llevó a cabo de enero de 2020 a enero de 2021, a la vez se se fue enfatizando en la elaboración del análisis de la acción y el proceso, y de forma simultánea se continuó en la búsqueda y recolección de los datos emergentes y el análisis^10^ de la información. Se realizaron entrevistas semiestructuradas, de manera virtual, a través de la aplicación Zoom, previa firma del Término de Consentimiento Libre e Informado (TCLE).

Las entrevistas duraron entre 25 minutos y 2 horas, y todas fueron grabadas. De acuerdo con el marco metodológico adoptado en este estudio^10^, fueron construidos memorándums, que son similares a los diarios de campo, que contienen los pensamientos del investigador, registran las comparaciones y conexiones realizadas, e indican las preguntas y las direcciones a seguir.

Para el análisis de datos, se utilizó el recurso de software NVivo 12 para realizar las codificaciones 10. Así, se realizaron los tres tipos de codificación: inicial, focalizada y axial, hacia la definición de conceptos y posibles supuestos de la dimensión existencial del ser-en-el-mundo de la ERC, a partir del referencial teórico adoptado. La información del análisis fue almacenada en Mendeley Data^11^.

Este estudio fue aprobado primero por el Comité de Ética de la Universidad Federal de Rio Grande do Sul, bajo el Certificado de Evaluación y Aprobación Ética (CAAE) 23534719.7.0000.5347, y luego por el Comité de Ética del Hospital de Clínicas de Porto Alegre, bajo el o CAAE 23534719.7.3001.5327.

## Resultados

La muestra de profesionales de enfermería estuvo compuesta por siete enfermeros (ENF) y tres técnicos de enfermería (TENF), en la que hubo predominio del sexo femenino, sólo había un enfermero y un técnico de enfermería. En cuanto a la antigüedad en la institución, osciló entre 5 y 25 años, lo que refleja la experiencia del equipo de enfermería en el servicio de Nefrología. En cuanto a TRS, la muestra estuvo compuesta por dos enfermeras de hemodiálisis, dos enfermeras de diálisis peritoneal y tres enfermeras de trasplante renal. Todos los técnicos de enfermería actuaban en la TRS de hemodiálisis.

La muestra de pacientes (P) estuvo compuesta por 10 participantes, predominantemente mujeres, con solo tres pacientes hombres. Las edades oscilaron entre 26 y 66 años, con predominio de pacientes en la treintena, con cinco pacientes con edades entre 32 y 39 años, demostrando la afectación de la ERC en adultos jóvenes. El TRS prevalente fue la hemodiálisis, realizada a cuatro pacientes, seguida de tres pacientes en diálisis peritoneal y tres pacientes trasplantados renales. Sin embargo, llama la atención que, incluso los pacientes que en el momento de la entrevista estaban en otra modalidad de TRS, todos, en algún momento, fueron sometidos a hemodiálisis.

A continuación, se presentan las categorías, subcategorías y sus elementos estructurantes que permiten identificar elementos conceptuales existenciales presentes en el contexto del cuidado del ser-en-el-mundo de la ERC a la luz de la gran teoría humanista de la enfermería^7^, como se muestra en el Cuadro 1.

**Cuadro 1:** Contexto de atención al ser-en-el-mundo con ERC

Categoría	Subcategoría	Elementos Estructurantes	Elemento Conceptual
		Adaptación a la realidad	Concepto
Contexto de	Ambiente	Establecer un espacio de convivencia	Concepto
cuidado			
		Convertirse en rutina	Concepto
	Temporalidad	Pasar el tiempo	Presupuesto

## Discusión

El concepto es entendido como una palabra o frase que resume un fenómeno, como una idea, observación o experiencia^12^. Generalmente, se convierten en variables utilizadas en hipótesis que se contrastan en la investigación y en la producción de conocimiento. Como los significados conceptuales son dinámicos, deben ser definidos para un contexto específico en el que el investigador quiere centrar el término^13^. Los presupuestos, por su parte, son elementos conceptuales, entendidos como creencias preconcebidas y aceptados como verdades, y por lo tanto no se prueban empíricamente, pero generalmente se confirman. Pueden basarse en conocimientos aceptados o en creencias y valores personales, además de ser cuestionados filosóficamente^12^.

En el contexto del cuidado, es importante comprender que éste atañe no sólo al espacio físico en el que se da, sino, sobre todo, a las interrelaciones que en él se construyen a lo largo del tiempo y que reflejan la forma en que el cuidado es los seres siguen existiendo en este mundo de ERC^8^.

### Subcategoría Ambiente

### Concepto - Adaptación a la Realidad

Como se muestra en el Cuadro 1, la adaptación a la realidad es un elemento estructurante de la categoría contexto de cuidado, entendida como una estrategia del ser-en-el-mundo de la ERC para continuar existiendo, inherente a la condición en que se encuentra el ser-paciente y el ser-enfermero pasando a relacionarse en un ambiente que es dinámico.

La adaptación a la realidad se convierte en una actitud constante a descubrir en medio de las limitaciones que impone la ERC y que están influenciadas por la modalidad de TRS utilizada por el ser- paciente, por el tiempo y por el mundo exterior en el que se encuentra. Esta elección de TRS permite diferentes posibilidades de experiencias, sin embargo, también exige renuncias, lo que muchas veces compromete la adhesión del ser-paciente a las diferentes TRS^14^, condición que es sustentada en los discursos de los participantes:

*“Si me voy a hacer diálisis peritoneal en casa, además de tener que tener un lugar específico para hacerlo, además de guardarlo, está todo ese cuidado, se va a infectar, entonces no me atienden durante la diálisis, así que pienso lo peor". (P3)*

*“Tuve que readaptarme a esta nueva vida [...] lo malo de la diálisis peritoneal es esto, estás solo, en el otro tenías amistad, hablabas, pero no tenía seguimiento médico. En este tengo, así que todo tiene pros y contras.” (P9)*

*“Trabajé durante 10 años en otro hospital, entonces la atención allí, la enseñanza era diferente, el tipo de paciente era diferente. Entonces era otra realidad para los pacientes que tenemos aquí. Aquí tenemos que adaptarnos a su realidad”. (ENF3)*

*“Ya he visto casos de pacientes que estaban en DP, tenían peritonitis severa, se hacían hemodiálisis y les gustaba, que al principio es aburrido, tener que ir al hospital, pero luego se acostumbran a ese ambiente y les resulta un lindo ambiente y like y comunica esto, bah, chévere, aquí me divierto más.” (ENF4)*

*“Ahora con el COVID-19, este tema será mucho más fácil en línea, será una gran experiencia de aprendizaje para todos, enfermeros y pacientes. En estos días estuve pensando en esto, en las alternativas de cuidado [...] hay mucha orientación para seguir, así que pensé, hagamos un video y lo ponemos en televisores, vamos a tener que usar esta opción. Así que estamos comprometidos con este otro cambio allí”. (ENF5)*

En este sentido, se definió el concepto de “Adaptar la realidad”, entendido como una estrategia para que el ser-en-el-mundo de la ERC siga existiendo, como una actitud constante por descubrir en medio de las limitaciones que impone la ERC y que son influenciados por la modalidad de TRS elegida por el ser-paciente, por el tiempo y por el ambiente, que es dinámico^8^.

### Concepto - Establecer un Espacio de Convivencia

Establecer un espacio de convivencia es también un elemento estructurante, entendido como resultado de la acción de adaptar la realidad, según el Cuadro 1. Se entiende, en la dimensión existencial, donde el yo es propriamente um “estar con o ser con”, y ese “estar con” se relaciona con otros “yoes” que forman “mi” mundo circundante^15^. En este contexto, los seres de mi mundo se distinguen de los objetos en que “también están ahí conmigo”; además, este “con” y este “también” son existenciales. Comparto el mundo “con los demás”, en el sentido de que uno no se distingue de los demás entre los cuales también está ^4^^,^^8^. Así, el “ser-en-el-mundo”, en un mundo en común, posibilita al ser existir en común con los demás, constituyendo un espacio de convivencia^4^.

Así, el ambiente de las TRS es el lugar que permite al ser-paciente y al ser-enfermero encontrarse con presencia en su totalidad, compartiendo experiencias en un espacio personalizado, vivo y, por lo tanto, dinámico. Los seres-en-el-mundo de la ERC desarrollan un sentido de pertenencia a este lugar, resignificando este entorno, a lo largo del tiempo, como un espacio de eventos sociales, entre familiares, donde es posible convivir y relacionarse con otros seres, que se evidencia en los discursos de los participantes^8^.

*“El ambiente es agradable porque no es un ambiente así con absoluto silencio. Técnicos, personas juegan entre sí, todo dentro de un volumen. Hay vida dentro del salón, no es algo muerto. Una vida normal funciona allí. [...] en el salón [...] veo vida, veo movimiento.” (P6) “Veo que para algunos es como una actividad social, tener que hacerse hemodiálisis es una actividad social, tienen más integración con nosotros, más interacción con nosotros que con su propia familia”. (ENF2) “Yo ya tuve un paciente que se hizo un trasplante, estaba feliz de tener el trasplante, pero cuando se dio cuenta de que no iba a venir a diálisis, no iba a ver a sus amigos 3 veces por semana, informó: yo estaba muy triste cuando me di cuenta que ya no los vería todas las semanas [...]. Porque a veces están muy solos [...]". (ENF4)*

*“Hay algunos que creo que su vida social es la diálisis. Hay pacientes que van a diálisis y acaba siendo el salir de la rutina, hablar con los compañeros, tener nuestra atención. [...], hay una pareja que se terminó conociendo en diálisis, son nuestros viejos allá, y se conocieron y ahora están comprometidos". (TENF3)*

Al explorar el diálogo de enfermería tal como es vivido en el mundo real, se hace evidente el factor ambiental. Cuando pensamos en equipamientos para el cuidado de la salud, “entorno” puede ser sinónimo de cosas como una cama, salas de espera, área de visitas, un lugar solitario, entre otros. Por supuesto, el entorno físico, ya sea en un hospital, una clínica o en cualquier otro lugar de la comunidad, puede servir para facilitar o impedir este diálogo. Sin embargo, la experiencia personal del ambiente puede ser aún más importante^7^.

El ser-enfermero y el ser-paciente pueden estar juntos en un mismo lugar y, aun así, uno puede sentir que pertenece a ese sitio mientras que el otro no, por lo tanto, el encuentro no se produce. Esto exige que el ser-enfermero comprenda el ser-paciente en su espacio de vida, vinculado al tiempo de vivir, en su aquí y ahora, refiriéndose a la interrelación existente entre ambiente y temporalidad en el contexto del cuidado^7^^,^^16^.

Así, se definió el concepto de “Establecer un espacio de convivencia” como resultado de la acción de adaptar la realidad, en la que es posible “ser con” o “estar con” otros seres-en-el-mundo de la ERC, entendiendo que ser significa existir en común con los demás, generando así un espacio seguro, personalizado, vivo y que permita compartir experiencias, convivencia^8^.

El concepto de “Ambiente” se definió como el contexto de cuidado que concierne no sólo al espacio físico en el que se da, sino que y, sobre todo, se refiere a las interrelaciones que en él se construyen a lo largo del tiempo y que se reflejan en la forma en que los seres seguen existiendo en el mundo. Es una especie de espacio vacío, un espacio personalizado o más propiamente, se refiere al lugar al que uno siente que pertenece o del cual forma parte^8^.

### Subcategoría Temporalidad

La temporalidad en el contexto del cuidado es inherente a la experiencia del tiempo y de la existencia resuelta, relativa al momento y al espacio en que se enraíza el cuidado. El “ser” es cuidado con anticipación de sí mismo, por lo tanto, no es una conducta particular y contingente del “ser” en relación consigo mismo, sino que define adecuadamente, en su unidad profunda, todas las determinaciones del ser. Así, el “ser” es cuidado mientras existe y, en su modo de “ser-en-el-mundo”, necesita ser descubierto^4^.

### Presupuesto - Pasar el tiempo

Pasar el tiempo, en la dimensión existencial, es un presupuesto de la condición de ser-en- el-mundo de la ERC, necesaria para que el ser-paciente y el ser-enfermero puedan develar nuevas posibilidades para trascender y seguir conviviendo hacia el futuro^8^.

Existencialmente, el hombre es entendido como un ser individual, necesariamente relacionado con otros hombres en el tiempo y el espacio. Cabe señalar, sin embargo, que este tiempo no siempre está relacionado y sincronizado con el tiempo cronológico, sino que es inherente al tiempo interno, único, vivido por los seres-en-el-mundo de la ERC, configurando la unicidad de cada ser, que tiene su propio ritmo para adaptarse a esta nueva realidad^4^^,^^7^^,^^15^, retratado en las declaraciones de los participantes.

*“Estas horas son interminables. Tomo la radio, tomo el móvil, escucho alguna noticia. A veces veo la televisión un rato. Sigo tratando de distraerme, incluso por los tiempos de las cosas sé que se acerca el final, porque escucho mucho”. (P6)*

*“El tiempo a veces era cruel, a veces era bonito, porque a veces el tiempo no pasaba, había días que no pasaba. Mirabas el reloj, pasaban 10 minutos, pasaban cinco minutos, y había días que pasaban naturalmente, que ni siquiera veías pasar. Así que este tema del tiempo era bastante complejo”. (P7)*

*“Es que hay varios tiempos, porque el paciente piensa que es mucho tiempo estar 4 horas en terapia, eso para él es un rato, para nosotros el tiempo pasa rápido, para él pasa muy lento”. (ENF5)*

### Concepto - Convertiese en Rutina

El pasar del tiempo permite que los seres se reúnan en este espacio, desarrollando recursos internos para vivir mejor. Así, tornandose en rutina, emerge como elemento estructurante de la subcategoría de la temporalidad, ligada a la temporalidad de la existencia del ser, inherente al pasado, presente y futuro, que debe ser comprendida singularmente en los seres-en-el-mundo de ERC, o sea, ser- paciente y ser-enfermero^7^^,^^15^.

Se entiende entonces, que al vivenciar el contexto de la ERC con su respectivo TRS, los seres de este mundo crean vínculos, se familiarizan con este ambiente y en el transcurso de su tiempo interno se adaptan a esta nueva realidad, buscando estrategias hacia su “ser-más” en este entorno, incorporándolo a su rutina. Es evidente, sin embargo, que la rutina es vivida de manera diferente por el ser-paciente y el ser-enfermero. Puede darse que el primero desarrolle una rutina para vivir mejor, siendo percebida como algo positivo, y, sin embargo, el segundo puede sentirse incómodo al repetir continuamente las mismas actividades^8^^,^^17^.

*“Hoy para mí es, esta mañana pasó rápido. Cuando vi que mis cuatro horas ya habían pasado, me acostumbré a esta rutina”. (P2)*

*“Es que yo leo cómics, o leo un libro, todo en la tablet, a veces hasta veo alguna serie que me descargo, así que está bien. Encontré esta manera de disfrutar mi tiempo allí” (P3)*

*“Se vuelve rutina. A veces apesta, porque es agotador, porque todos los días es lo mismo. La rutina es algo que me molesta a veces, no siempre, especialmente en hemodiálisis”. (ENF3)*

*“A medida que pasa el tiempo, buscan formas de engañar al tiempo. Veo que la gran mayoría se organiza. Los mayores ya conocen la rutina. Buscan la forma de mejorar, de aprovechar mejor su tiempo allí en el aula. En cuanto a nosotros [...] está bien, pero a veces cansa un poco, porque es algo repetitivo, entonces siempre es lo mismo”. (ENF6)*

Además, la temporalidad hace que el ser-paciente, después de un período de tiempo, manifieste muchas veces una actitud de propiedad de los objetos que forman parte del ambiente de cuidado, como la cama, la máquina de hemodiálisis o la diálisis peritoneal^7^^-^^8^^,^^18^^-^^19^, con la que el ser-paciente establece una relación de dependencia, en la que la máquina representa para él la vida.

Lo mismo ocurre en la relación entre ser-paciente y ser-enfermero, en la que, con el tiempo y la familiaridad, se desarrolla un sentimiento de pertenencia recíproca. El ser pertenece al lugar y el lugar pertenece al ser. En ese sentido, el diálogo vivido entre seres es estimulado por la conciencia que el ser enfermero, en postura empática, tiene no sólo con su propia experiencia del espacio sino también con la del ser-paciente^20^^-^^21^. Esta percepción del ambiente como espacio vivo, que está influenciado por el tiempo y las interacciones que en él se dan, así como la pertenencia recíproca, está presente en el discurso de algunos participantes.

*“Los técnicos están muy motivados, atienden muy bien al paciente, son muy competentes, pero cuando se topa con eso que interfiere en mi tiempo [...], si doy espacio ahora para que haga este profesional, o incluso adaptarnos [. ..], entonces superar esas aristas [...] muchas veces nos obliga a trabajar mucho con el equipo de enfermería que “siente” al paciente, en ese momento el paciente está ahí, como mi paciente, al igual que el paciente también, es mi técnico, es mi máquina, entonces a veces no lo compartimos muy bien.” (ENF5)*

*“Creo que un poco también es por qué, por la convivencia que tenemos desde hace mucho tiempo y varias veces a la semana, creo que eso crea una falsa ilusión de posesión, pero no una posesión posesiva, de querer a la persona para uno mismo, sino de control". (TENF2)*

Por lo tanto, el concepto de "convirtirse en rutina" se definió como resultante de la adaptación de la realidad ligada a la temporalidad del ser, que al experimentar el mundo de la ERC crea vínculos, se familiariza con el entorno y, en el transcurso de su tiempo interno, se adapta a esta nueva realidad, buscando estrategias para su “ser-más”^8^.

El concepto de “Temporalidad” se definió como el pasar del tiempo, pasado, presente y futuro, entendido como una tensión continua entre el ser y el mundo, entendiendo que hay una experiencia del tiempo interno del ser, donde reside la autoconciencia, y una del tiempo externo, inherente a las cosas del mundo, y que es en la experiencia de la temporalidad y de la existencia resuelta que radica el cuidado, que permite al ser desarrollar un sentimiento de pertenencia^8^.

En este sentido, la categoría Contexto de Cuidado es entendida como un ambiente que es un espacio vivo, dinámico, de interrelaciones entre seres-en-el-mundo de la ERC, quienes, en la temporalidad de cada uno, comparten experiencias, buscando adaptarse a la realidad para estar mejor en este mundo. En ese escenario, el cuidado se da cuando se establece un espacio de interacción social, que permite a los seres-en-el-mundo de la ERC buscar la pertenencia a ese medio, posibitar la intimidad, generar amistades, generar espacios de esparcimiento, estudio, y a veces, de sufrimiento y llanto, buscando permanecer en este mundo^8^ y transcender.

## Conclusiones

El presente estudio posibilitó comprender el contexto del cuidado en el mundo de la ERC desde la perspectiva de la teoría humanista de Enfermería, identificando a partir de los datos, la categoría Contexto del Cuidado, constituida por dos subcategorías, ambiente y temporalidad, de donde surgen los conceptos y el presupuesto siguientes, conceptos Ambiente, Adaptación a la Realidad, Establecer un espacio de convivencia, Temporalidad, Convertirse en rutina y el presupuesto Pasar el tiempo.

Se entiende que la enfermería, como ciencia teórico-práctica, necesita referenciales teóricos que orienten la práctica clínica hacia un cuidado que contemple las dimensiones biológica, espiritual y existencial del ser, haciendo más efectivo ese cuidado, en tanto busca al ser en su totalidad, considerando también el contexto donde ocurre ese cuidado, como el medio ambiente, que influye en la adaptación y adherencia al tratamiento.

Dicho esto, se entiende como una limitación de este estudio, la no realización de un modelo de atención con estos conceptos y presupuestos existenciales en el mundo de la ERC. En ese sentido, se recomienda realizar más estudios con el objetivo de identificar nuevos conceptos y presupuestos existenciales presentes en este contexto, y así establecer un modelo teórico/práctico de cuidado, así como desarrollar una teoría de medio rango en esta perspectiva existencial para orientar la atención a los pacientes en TRS.

Se espera que este estudio pueda contribuir a la práctica clínica en el contexto de la ERC, para una mirada más humanizada, atenta a la influencia del ambiente y la temporalidad en ese proceso de adaptación del ser-paciente al TSR de elección, haciéndolo posible generar alternativas para mejorar las condiciones ambientales, como la incorporación de nuevas prácticas terapéuticas, así como la mejora del ambiente físico hacia el cuidado individualizado, que rescate la singularidad de cada persona cuidada, lo cual está en consonancia con la esencia de la Enfermería.
